# Descrição das deformidades do quadril em pacientes de 5 anos de idade com síndrome congênita do vírus Zika

**DOI:** 10.1055/s-0045-1814121

**Published:** 2025-12-30

**Authors:** Amnuay Kleebayoon, Viroj Wiwanitkit

**Affiliations:** 1 Consultor Acadêmico Particular, Samraong, Camboja; 2 Departamento de Medicina Comunitária, D Y Patil Medical College, Hospital and Research Centre, Dr. D Y Patil Vidyapeeth (Deemed to be University), Pune, India

Prezado Editor,

O objetivo do estudo “Descrição das deformidades do quadril em pacientes com síndrome congênita do vírus Zika aos 5 anos de idade: Um estudo transversal” 1 foi analisar anomalias comuns do quadril em crianças com mais de cinco anos de idade com síndrome congênita do vírus Zika (SCZ) por meio de exames clínicos e radiográficos. Embora o estudo forneça informações úteis para uma população específica de pacientes, apresenta limitações metodológicas e estatísticas que devem ser cuidadosamente avaliadas. Um exemplo é o desenho transversal e retrospectivo do estudo, que pode introduzir viés de seleção e limitar a capacidade de discernir com precisão causa e efeito entre as variáveis.

O tamanho da amostra de apenas 62 pacientes pode ser insuficiente para análises estatísticas complexas, particularmente quando estratificadas por nível de GMFCS (do inglês, Gross Motor Function Classification System), resultando em menos indivíduos por grupo e menor precisão e poder estatístico. Além disso, não foram fornecidos intervalos de confiança nem controles para variáveis de confusão, como outros históricos médicos ou fatores nutricionais, que pudessem influenciar o desenvolvimento estrutural do quadril. Embora os dados de comparação estivessem disponíveis, as relações entre variáveis como índice de Reimers (IR), índice acetabular (IA) e ângulo colo-diáfise femoral (ACDF) e tenotomia não esclareceram como as covariáveis foram controladas ou pareadas, o que limitou a interpretação dos resultados.

As perguntas para inspirar a discussão acadêmica incluem: A associação entre o GMFCS e as mudanças radiográficas reflete o desenvolvimento genuíno da doença ou é apenas o resultado de diferenças nos hábitos de movimento entre os grupos? A ausência de diferença na ACDF entre os grupos é clinicamente significativa? Está relacionado às limitações de tamanho da amostra? Outro achado intrigante é que, embora a tenotomia aumente a extensão da perna, não afeta os valores radiográficos, o que implica que as terapias cirúrgicas devem ser avaliadas para mais do que simplesmente resultados clínicos de curto prazo.

Estudos futuros devem incluir estudos de coorte para avaliar alterações radiográficas e mobilidade a longo prazo. Além disso, os tamanhos das amostras devem ser maiores e os dados de vários locais devem ser incorporados para melhorar a generalização dos resultados. Novas tecnologias, como a modelagem 3D de imagens radiográficas ou a inteligência artificial para análise de imagens, devem ser empregadas para determinar as ligações intrincadas entre características anatômicas e resultados clínicos, permitindo um atendimento individualizado mais eficaz a pacientes com SCZ.
